# How Italian respiratory physiotherapists have faced and are facing the coronavirus disease 2019 pandemic

**DOI:** 10.1186/s40945-020-00086-8

**Published:** 2020-08-03

**Authors:** Marta Lazzeri

**Affiliations:** 1 Department of Cardiothoracic and Vascular Surgery, ASST Grande Ospedale Metropolitano, Milan, Niguarda Italy; 2 Associazione Riabilitatori dell’Insufficienza Respiratoria, Spinal Unit, ASST Grande Ospedale Metropolitano, Milan, Niguarda Italy

Dear Editor:

The whole world is facing an unknown disease that often manifests itself in a serious and deadly form: the coronavirus disease 2019, or COVID-19, which is caused by viral infection (SARS-CoV-2) and presents with severe acute respiratory syndrome. Symptoms of COVID-19 can vary. The most frequent are dry cough, asthenia, fever, and in more severe cases (approximately 25%) pneumonia characterized by bilateral interstitial infiltrates with hypoxic respiratory failure (i.e., acute respiratory distress syndrome; ARDS) after serious alteration of the ventilation/perfusion ratio (VA/Q). It is associated with a systemic inflammatory response that can induce microcirculation alterations, arrhythmia, myocarditis, renal and liver failure. Non-invasive intervention such as high-flow nasal oxygen therapy, continuous positive airway pressure, and non-invasive mechanical ventilation, aimed to correct hypoxemia and to reduce the work of breathing, can help to avoid endotracheal intubation with its potential complications and wasting effects on outcomes or survival. However, these interventions must be managed in appropriate settings with a careful monitoring, since rapid and unexpected worsening often occurs, necessitating immediate endotracheal intubation. In the acute phase, having specialized health professionals with specific respiratory skills has been critical. Where present, respiratory physiotherapists (RPh) made a great contribution by working together with other health professionals in assisting patients, providing “quick basic training sessions” about respiratory care for non-specialist healthcare professionals, locating and assessing all kinds of equipment (ventilators, interfaces, oxygen delivery systems, among others) to assist patients with respiratory failure and implementing early intervention with those patients in healthier conditions. The expertise previously gained in the treatment of acute and chronic respiratory failure conditions due to different etiopathogeneses (such as fibrosis, exacerbation of chronic obstructive pulmonary disease, interstitial lung diseases, difficult to wean post-acute patients) was extremely valuable, because the clinical functional manifestations of many of the aforementioned conditions are not different to those deficits observed in COVID-19.

Knowledge about the oxygen delivery systems and interfaces, non-invasive mechanical ventilation, weaning from assisted ventilation and tracheostomy, airway clearance strategies and techniques, early mobilization and posture variation is applied every day to optimize ventilation, to improve gas exchanges, to prevent secondary impairments and possible post-acute sequelae, and even to reduce the intensive care unit and hospital stay. Moreover, when clinical conditions allow it, all actions to promote the patient autonomy in respiration, exercise tolerance and mobility in the daily life activity and other basic functions including coughing, phonation, and swallowing are ensured.

Many hospital departments have been converted to admit COVID-19 patients: intensive and respiratory sub-intensive care beds expaned exponentially; pre-triage to access emergency rooms and separate routes for COVID+ and COVID- patients were established; scheduled activities, both for outpatients and for medical/surgical hospitalization were suspended, except for emergencies. Some hospitals planned physiotherapists’ work schedules to cover a 12-day service (morning and afternoon), and re-organized physiotherapist teams (also with newly hired physiotherapists) to adequately cover weekends and holidays. In addition, all therapeutic interventions, distinguishing between essential and non-essential services, have been reprogrammed.

The telephone counseling or telemonitoring and tele-rehabilitation interventions have been established for children, elderly people and patients with chronic diseases. Unfortunately, and very quickly, a number of posts appeared on social networks and on other websites offering “new” respiratory therapies actually devoid of a scientific basis, or suggesting obsolete “respiratory exercises” protocols widely rejected in literature many years ago; some of these procedures are useless, if not even risky, for patients with post-acute respiratory failure. Physiotherapists working in COVID-19 units urgently sought out information about protective measures to be adopted and correct physiotherapeutic interventions to be used in different situations. Despite the absolute novelty of the pathology and the lack of scientific evidence, based on the available knowledge of physiopathology and respiratory care strategies, in collaboration with other scientific societies (Associazione Italiana Fisioterapisti -AIFI-, Associazione Italiana Pneumologi Ospedalieri -AIPO-, Società Italiana Pneumologia -SIP-, European Respiratory Society-ERS-), documents have been published in open-access journals and platform aimed to promote safe use of personal protective equipment (PPE) and to support the decision process in the management of COVID-19 patients in the acute and post-acute phases [1–4].

Now hospitalizations are decreasing and many patients are discharged: assessment tools are being prepared to measure respiratory, cardiological, neuromotor, cognitive, and emotional impairment, and to stratify patients in order to direct more impaired individuals to rehabilitation facilities, and to discharge those patients who recovered at least partial independence to centralized quarantine facilities or to home. As subjects affected by COVID-19 can be contagious for a long time, even after two consecutive negative swabs, remote management and follow-up for at least after 3 months after discharge are ongoing. After 3 months of experience with COVID-19, data show typical neuromuscular sequelae and critical illnesses seen mostly in ARDS survivors after being bedridden in intensive care units, as well as severe complications affecting the cardio-respiratory system, even in patients with few or non-symptoms. Already, control CT scans performed before discharge often show signs of pulmonary fibrosis, lung damage that can seriously limit functional capacity and quality of life. Given the novelty of COVID-19 pneumonia, the impact of permanent lung injury after recovery is unknown. After Middle East respiratory syndrome coronavirus infection (which is similar to COVID-19 in some ways), about 1/3 of the patients showed signs of pulmonary fibrosis at radiological follow-up [5, 6].

Scientific societies have recommended low-intensity exercise training (< 3.0 METs), with a gradual increase in workload based on symptoms (dyspnea and/or fatigue Borg score ≤ 3–4) and objective measures such as oxygen saturation level and heart rate.

A careful monitoring by an expert healthcare professional is mandatory, as many patients have unexpected reactions to physical activity and exercise. Severe desaturation or marked tachycardia occurs also in non-symptomatic patients. In addition, non-comorbid young patients recovered from COVID-19, without having been intubated or sedated during hospitalization, show severe fatigue for weeks after discharge. Many of these people might require a long-term monitoring and management related to functional outcomes, such as fatigue, weakness and pre-existing disabilities (e.g. a program of aerobic exercises, strength and balance training, strategies for energy conservation, functional rehabilitation).

During this pandemic (and for a long time going forward) healthcare workers need to wear protection that limit their movement, that scar their faces, that increase their fatigue and sweat for many hours every day, and “hide” them to patients. The emotional load is great assisting lonely people, far from their most loved ones, undergoing life-saving procedures. People die without being able to receive a last hug, a caress from their family, or one last greeting “We love you. We will not forget you,” They can only receive a “smile” through our eyes as the last comfort.

In addition, there is the grief for relatives and friends who have fallen ill and died, and there is the fear of being infected and infecting our families, but also the awareness and the great pride to be key actors, together with all the other health professionals, with our valuable contribution in this awful war. Now, more than ever before, physiotherapists are called to action. Nevertheless, we need to widen our competences to answer to these new health needs, we need to rewrite organizational models in the acute settings, and to empower our role in the community.

Once again, the history of this pandemic reminded us of the vital role of the scientific community, with its communication channels, such as peer-reviewed scientific journals. All health professionals are full-fledged members of this scientific community, each with a privileged viewpoint, and each one with the potential to acquire and interpret unique and indispensable information to make the overall picture more complete. Now that the emergency gradually makes room for a new normality, it will be essential to continue collecting data, documenting successes and failures of our work, during these and the next months, especially through the publication of empirically derived observations and results. There is much to be learned from this experience, and it will make us more ready to face future challenges, including those pertaining to physiotherapy and pulmonary rehabilitation.

## Data Availability

Not applicable.
